# Supplementary material for: Prevalence, Characteristics, Management and Outcomes of Patients with Heart Failure with Preserved, Mildly Reduced, and Reduced Ejection Fraction in Spain
Source: J Clin Med. 2022 Sep 2;11(17):5199. doi: 10.3390/jcm11175199 (PMC9456780; doi:10.3390/jcm11175199)
Supplement: Supplementary file 1 [file jcm-11-05199-s001.zip › jcm-1817921-supplementary.pdf]

Supplementary table S1. Definition of the variables (codes).

ICD-9	ICD-10	Description
402.01, 402.11, 402.91	I11.0	Hypertensive heart disease with heart failure
404.01, 404.11, 404.91	I13.0	Hypertensive heart and renal disease with (congestive) heart failure
404.03, 404.13, 404.93	I13.2	Hypertensive heart and renal disease with both (congestive) heart failure and renal failure
429.4, 997.1	I97.1	Other functional disturbances following cardiac surgery - Heart failure
428.0	I50.0	Congestive Heart failure
428.1	I50.1	Left ventricular failure, unspecified
428.2	I50.2	Systolic heart failure
428.3	I50.3	Diastolic heart failure
428.4	I50.4	Combined systolic and diastolic heart failure
428.9	I50.9	Heart failure, unspecified
238.72, 238.73, 280.0, 280.8, 280.9, 281.0, 281.1, 281.2, 281.3, 281.4, 281.8, 281.9, 282.0, 282.1, 282.2, 282.3, 282.5, 282.7, 282.8, 282.9, 282.40, 282.43, 282.44, 282.45, 282.46, 282.49, 282.61, 282.62, 82.68, 282.69, 283.0, 283.2, 283.9, 283.11, 283.19, 284.0, 284.8, 284.81, 284.89, 284.9, 285.0, 285.1, 285.8, 285.9, 285.22, 285.29	D50.0, D50.1, D50.8, D50.9, D51.0, D51.1, D51.2, D51.3, D51.8, D51.9, D52.0, D52.1, D52.8, D52.9, D53.0, D53.1, D53.2, D53.8, D53.9, D55.0, D55.1, D55.2, D55.3, D55.8, D55.9, D56.0, D56.1, D56.2, D56.3, D56.4, D56.8, D56.9, D57.0, D57.1, D57.2, D57.3, D57.8, D58.0, D58.1, D58.2, D58.8, D58.9, D59.0, D59.1, D59.2, D59.3, D59.4, D59.5, D59.6, D59.8, D59.9, D60.0, D60.1, D60.8, D60.9, D61.0, D61.1, D61.2, D61.3, D61.8, D61.9, D62, D63.0, D63.8, D64.0, D64.1, D64.2, D64.3, D64.4, D64.8, D64.9, D46.0, D46.1, D46.2, D46.4, D46.5	Anemia

Supplementary table S2. Incidence rates of HF.

	Number of patients with HF[1]	Person years[2]	Incidence per 100 person-years	95% confidence interval
**All HF patients**				
Overall	20,063	6,204,285	0.32	0.32-0.32
2013	2,680	978,454	0.27	0.27-0.27
2014	2,669	874,485	0.31	0.30-0.31
2015	2,708	878,662	0.31	0.30-0.31
2016	3,155	940,391	0.34	0.33-0.34
2017	2,971	914,202	0.32	0.32-0.33
2018	3,460	924,621	0.37	0.37-0.38
2019*	2,420	693,470	0.35	0.35-0.35
**HFrEF**				
Overall	8,745	6,199,443	0.14	0.14-0.14
2013	1,230	977,850	0.13	0.12-0.13
2014	1,109	873,792	0.13	0.12-0.13
2015	1,198	878,021	0.14	0.13-0.14
2016	1,430	939,629	0.15	0.15-0.15
2017	1,340	913,531	0.15	0.14-0.15
2018	1,373	923,811	0.15	0.15-0.15
2019*	1,065	692,808	0.15	0.15-0.15
**HFpEF**				
Overall	5,266	6,197,943	0.09	0.08- 0.09
2013	672	977,633	0.07	0.07-0.07
2014	743	873,660	0.09	0.08-0.09
2015	718	877,747	0.08	0.08-0.08
2016	785	939,411	0.08	0.08-0.08
2017	762	913,285	0.08	0.08-0.08
2018	923	923,600	0.10	0.09- 0.10
2019*	663	692,607	0.10	0.09 -0.10
**HFmrEF**				
Overall	1,026	6,196,141	0.02	0.01-0.02
2013	85	977,407	0.01	0.01-0.01
2014	150	873,404	0.01	0.01-0.01
2015	193	877,546	0.02	0.02-0.02
2016	120	939,108	0.01	0.01-0.01
2017	161	913,015	0.02	0.02-0.02
2018	209	923,318	0.02	0.02-0.02
2019*	108	692,343	0.02	0.01- 0.02
**HFuEF**				
Overall	5,026	6,197,842	0.08	0.08-0.08
2013	693	977,673	0.07	0.07-0.07
2014	667	873,627	0.08	0.07-0.08
2015	599	877,732	0.07	0.07-0.07
2016	820	939,399	0.09	0.08-0.09
2017	708	913,220	0.08	0.08-0.08
2018	955	923,590	0.10	0.10-0.10
2019*	584	692,601	0.08	0.08-0.08

Abbreviations: HF = Heart failure; HFmrEF = Heart failure with mildly reduced ejection fraction; HFpEF = Heart Failure with preserved ejection fraction; HFrEF = Heart failure with reduced ejection fraction; HFuEF = Heart Failure with unspecified ejection fraction.

1. Number of patients who have a new qualifying HF diagnosis during the year.

2. Total person time contributed by all adults from the start of the year until the earliest of a qualifying HF diagnosis, death, the study end date, and loss to follow-up.

*year 2019 includes until September.

Supplementary table S3. Annual prevalence of HF.

	No. of Patients with HF[1] (HFrEF ≤40%)	Total number of patients[2]	Prevalence (%)	95% confidence interval (%)
**All HF patients**				
Overall	43,578	1,865,061	2.34	2.32 - 2.36
2013	20,628	995,381	2.07	2.04 - 2.10
2014	20,634	902,303	2.29	2.26 - 2.32
2015	21,209	911,618	2.33	2.30 - 2.36
2016	21,400	989,220	2.16	2.13 - 2.19
2017	22,060	971,683	2.27	2.24 - 2.30
2018	23,344	956,461	2.44	2.41 - 2.47
2019*	23,931	1,009,806	2.37	2.34 - 2.40
**HFrEF**				
Overall	20,901	1,865,061	1.12	1.10 - 1.14
2013	10,485	995,381	1.05	1.03 - 1.07
2014	10,082	902,303	1.12	1.10 - 1.14
2015	10,304	911,618	1.13	1.11 - 1.15
2016	10,396	989,220	1.05	1.03 - 1.07
2017	10,727	971,683	1.10	1.08 - 1.12
2018	11,166	956,461	1.17	1.15 - 1.19
2019*	11,828	1,009,806	1.17	1.15 - 1.19
**HFpEF**				
Overall	16,963	1,865,061	0.91	0.90 - 0.92
2013	7,661	995,381	0.77	0.75 - 0.79
2014	7,949	902,303	0.88	0.86 - 0.90
2015	8,183	911,618	0.90	0.88 - 0.92
2016	8,246	989,220	0.83	0.81 - 0.85
2017	8,455	971,683	0.87	0.85 - 0.89
2018	9,027	956,461	0.94	0.92 - 0.96
2019*	9,083	1,009,806	0.90	0.88 - 0.92
**HFmrEF**				
Overall	1,852	1,865,061	0.10	0.10 - 0.10
2013	866	995,381	0.09	0.08 - 0.10
2014	894	902,303	0.10	0.09 - 0.11
2015	875	911,618	0.10	0.09 - 0.11
2016	903	989,220	0.09	0.08 - 0.10
2017	982	971,683	0.10	0.09 - 0.11
2018	1,058	956,461	0.11	0.10 - 0.12
2019*	1,041	1,009,806	0.10	0.09 - 0.11
**HFuEF**				
Overall	3,862	1,865,061	0.21	0.20 - 0.22
2013	1,616	995,381	0.16	0.15 - 0.17
2014	1,709	902,303	0.19	0.18 - 0.20
2015	1,847	911,618	0.20	0.19 - 0.21
2016	1,855	989,220	0.19	0.18 - 0.20
2017	1,896	971,683	0.20	0.19 - 0.21
2018	2,093	956,461	0.22	0.21 - 0.23
2019*	1,979	1,009,806	0.20	0.19 - 0.21

Abbreviations: HF = Heart failure; HFmrEF = Heart failure with mildly reduced ejection fraction; HFpEF = Heart Failure with preserved ejection fraction; HFrEF = Heart failure with reduced ejection fraction; HFuEF = Heart Failure with unspecified ejection fraction.

1. Number of patients who had a qualifying HF diagnosis before the beginning of the year.

2. All adult patients alive and enrolled as of the beginning of the calendar year and who have been continuously enrolled during the entire year prior.

*year 2019 includes until September.

Supplementary table S4. Event Rates of myocardial infarction, stroke and HF hospitalization in the prevalent HF Cohort (2016 cohort only).

	All HF (N=21297)			HFrEF (N=10323)			HFmrEF (N=903)			
	Patients with outcome (n)	Total person-years	Rate*	Patients with Outcome (n)	Total person-years	Rate*	Patients with Outcome (n)	Total person-years	Rate*	p-value (versus HFrEF)
MI	761	17.495	43.5 (31.2 - 60.4)	429	8.492	50.5 (33.5-75.5)	32	746	42.9 (9.4-174.3)	0.707
Stroke	613	17.495	35.0 (23.1 - 52.7)	367	8.492	43.2 (26.7-69.2)	14	746	18.8 (1.1-243.6)	0.034
HF hospitalization	4051	17.495	231.6 (218.8 - 244.8)	2269	8.492	267.2 (249.4-285.8)	144	746	193 (136.8-265.1)	<0.001

	HFpEF (N=8225)				HFpEF (50 to <60) (N=2995)			HFpEF (≥60) (N=5230)			HFuEF (N=1846)		
	Patients with outcome (n)	Total person-years	Rate*	p-value (versus HFrEF)	Patients with outcome (n)	Total person-years	Rate*	Patients with outcome (n)	Total person-years	Rate*	Patients with outcome (n)	Total person-years	Rate*
MI	238	6.737	35.3 (18.3-67.1)	<0.001	83	2.455	33.8 (11.2-97.7)	155	4.283	36.2 (16.3-78.5)	62	1.519	40.8 (12.8-122.4)
Stroke	191	6.737	28.3 (12.6-62.7)	<0.001	75	2.455	30.5 (9.1-97.8)	116	4.283	27.1 (9.5-75)	41	1.519	27 (5.1-129.9)
HF hospitalization	1332	6.737	197.7 (177.2-219.9)	<0.001	477	2.455	194.3 (161.3-232.2)	855	4.283	199.6 (174.2-227.7)	306	1.519	201.5 (160.3-250)

Abbreviations: CI = Confidence interval; HF = Heart failure, HFmrEF = Heart failure with mildly reduced ejection fraction; HFpEF = Heart Failure with preserved ejection fraction; HFrEF = Heart failure with reduced ejection fraction; HFuEF = Heart Failure with unspecified ejection fraction; MI = Myocardial infarction.

*Rate per 1,000 person-years (95% confidence interval)
